# Bellidifolin ameliorates isoprenaline-induced cardiac hypertrophy by the Nox4/ROS signalling pathway through inhibiting BRD4

**DOI:** 10.1038/s41420-023-01563-2

**Published:** 2023-08-01

**Authors:** Dingyan Zhou, Weizhe Liu, Juanjuan Zhang, Yucui Dong, Jiangli Wu, Yu Zhang, Cheng Dai, Tingting Zhang, Gaoshan Yang, Yue Zhang, Aiying Li

**Affiliations:** 1 Department of Biochemistry and Molecular Biology, College of Basic Medicine, Hebei University of Chinese Medicine, Shijiazhuang, China; 2 Hebei Higher Education Institute Applied Technology Research Center on TCM Formula Preparation, Shijiazhuang, China; 3 Hebei Key Laboratory of Chinese Medicine Research on Cardio-Cerebrovascular Disease, Shijiazhuang, China; 4 Department of Technology, Hebei University of Chinese Medicine, Shijiazhuang, China

**Keywords:** Cardiac hypertrophy, Cardiac hypertrophy

## Abstract

To date, there is no effective therapy for pathological cardiac hypertrophy, which can ultimately lead to heart failure. Bellidifolin (BEL) is an active xanthone component of *Gentianella acuta* (*G. acuta*) with a protective function for the heart. However, the role and mechanism of BEL action in cardiac hypertrophy remain unknown. In this study, the mouse model of cardiac hypertrophy was established by isoprenaline (ISO) induction with or without BEL treatment. The results showed that BEL alleviated cardiac dysfunction and pathological changes induced by ISO in the mice. The expression of cardiac hypertrophy marker genes, including ANP, BNP, and β-MHC, were inhibited by BEL both in mice and in H9C2 cells. Furthermore, BEL repressed the epigenetic regulator bromodomain-containing protein 4 (BRD4) to reduce the ISO-induced acetylation of H3K122 and phosphorylation of RNA Pol II. The Nox4/ROS/ADAM17 signalling pathway was also inhibited by BEL in a BRD4 dependent manner. Thus, BEL alleviated cardiac hypertrophy and cardiac dysfunction via the BRD4/Nox4/ROS axes during ISO-induced cardiac hypertrophy. These findings clarify the function and molecular mechanism of BEL action in the therapeutic intervention of cardiac hypertrophy.

## Introduction

Pathological cardiac hypertrophy is usually induced by pressure overload and leads to heart failure, which is a prevalent cause of morbidity and mortality worldwide [1, 2]. Cardiac hypertrophy is accompanied by enlarged cardiomyocytes, increased cardiac weight, and abnormal cardiac structure [3]. The expression of several marker genes, such as atrial natriuretic peptide (ANP), brain natriuretic peptide (BNP), and β-myosin heavy chain (β-MHC), leads to this pathological process [4]. Moreover, epigenetic modifications regulate cardiac hypertrophy by changing genome stability and gene expression via the methylation and acetylation regulation of histone proteins [5]. Metabolic changes and certain signalling pathways, such as the AMPK, mTOR, and Hippo pathways are also involved in this pathogenesis [6, 7]. Despite advancements in medical science, cardiac hypertrophy has not been effectively prevented or ameliorated to date. Research on effective drugs and relevant mechanisms to attenuate pathological hypertrophy is therefore of great significance.

Bromodomain-containing protein 4 (BRD4) plays a key role in cardiovascular diseases such as cardiac hypertrophy, infarction, and fibrosis as an epigenetic regulator [8]. BRD4 recognises histone H3K9ac and binds specific sequential regions of target genes. Then, H3K122 is acetylated by BRD4, resulting in nucleosome depolymerisation [9]. Moreover, BRD4 promotes the phosphorylation of RNA Pol II through the recruitment of the p-TEFb complex, which ultimately promotes the transcription of target genes [10]. BRD4 regulates a variety of biological processes, such as mitochondrial balance, cell proliferation, apoptosis, and inflammation [11–13]. Among downstream molecules of BRD4, NADPH oxidase 4 (Nox4) is the major source of ROS in the heart [14]. Previous studies have shown that Nox4 promotes cardiac hypertrophy via the ROS/ADAM17 pathway [15, 16]. BRD4 inhibition reduces Nox4 transcriptional activity and ROS production [17]. BRD4 has gradually been recognised as a target for epigenetic regulation in heart disease. However, there is no effective drug for reducing BRD4 function is on the market.

*Gentianella acuta* (*G. acuta*) is a herb of the Gentianaceae family that exhibits many pharmacological activities, such as reducing pain and inflammation, and shows antineoplastic activity [18–20]. Moreover, *G. acuta*, known as “Wenxincao” in Mongolia, China, is generally used to treat heart disease [21]. Our previous studies showed that *G. acuta* attenuated TAC-induced cardiac remodelling in rats [22]. Myocardial fibrosis and myocardial infarction induced by isoproterenol were also ameliorated by *G. acuta*, which inhibited the NF-κB signalling pathway [23, 24]. Bellidifolin (BEL) is a xanthone compound extracted from *G. acuta* that protects against cardiac fibrosis by repressing TGF-β1/Smad signalling pathway activation [25]. However, the regulatory function and mechanism of BEL action in myocardial cells during cardiac hypertrophy formation have not been studied. In this study, we explored the therapeutic effect of BEL on cardiac hypertrophy and elucidated the mechanism by which BEL confers myocardial protection through the BRD4/Nox4 pathway. The results show that BEL is a potential therapeutic agent for attenuating cardiac hypertrophy.

## Results

### BEL attenuated ISO-induced cardiac function abnormalities in mice

**Fig. 1:**
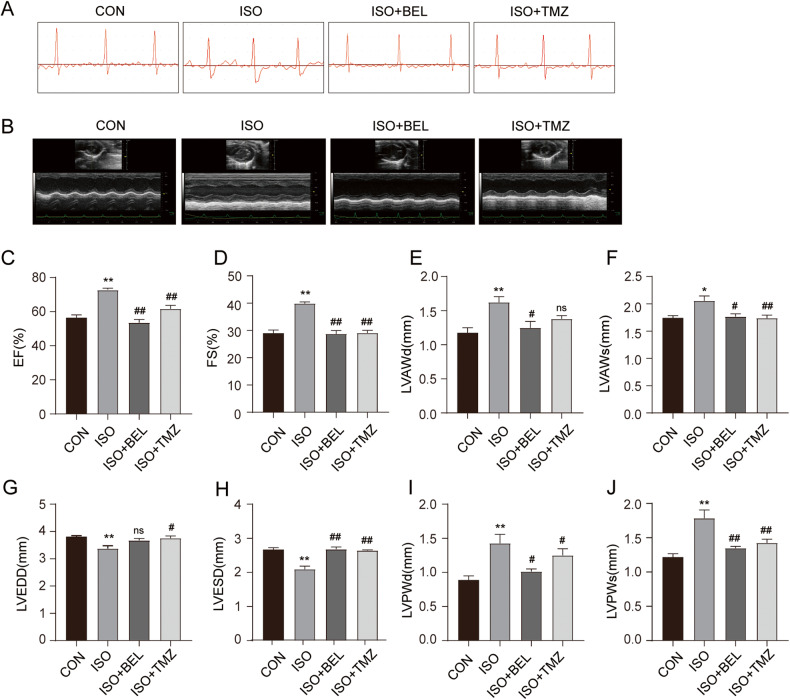
BEL improved cardiac function in ISO-treated mice. **A** Representative images of ECG tracings in the different groups. **B** Representative images of ultrasound in different groups. **C**–**J** Changes in EF, FS, LVAWd, LVAWs, LVEDD, LVESD, LVPWd, and LVPWs in the different groups (*n* = 6). Data are shown as the mean ± SEM. Significance: *p < 0.05 vs CON group; **p < 0.01 vs CON group; # *p* < 0.05 vs ISO group; ## *p* < 0.01 vs ISO group; ns no significant difference.

Cardiac function was assessed in mice with or without BEL treatment after ISO-induced cardiac hypertrophy via electrocardiography and echocardiography. The electrocardiography results showed that the ST segment was excessively depressed by ISO compared with the CON group (Fig. 1A). BEL treatment effectively improved these abnormalities. Echocardiography was also performed (Fig. 1B). The results showed that BEL and TMZ inhibited the increases in the ejection fraction and fractional shortening levels induced by ISO (Fig. 1C, D). The ISO-induced increases in LV diastolic anterior wall thickness (LVAWd) and LV systolic anterior wall thickness (LVAWs) were abolished by BEL treatment (Fig. 1E, F). However, the level of LVAWd was not significantly changed by TMZ treatment. In addition, the levels of LV end-diastolic diameter (LVEDD) and LV end-systolic diameter (LVESD) were significantly reduced by ISO (Fig. 1G, H). BEL treatment significantly increased the LVESD level with no obvious change in LVEDD compared with these levels in the ISO group. Furthermore, LV diastolic posterior wall thickness and LV systolic posterior wall thickness, which were increased by ISO induction, were reduced by BEL and TMZ (Fig. 1I, J). The data demonstrated that BEL improved cardiac function disorders in ISO-treated mice.

### BEL alleviated cardiac pathological disorders induced by ISO in mice

**Fig. 2:**
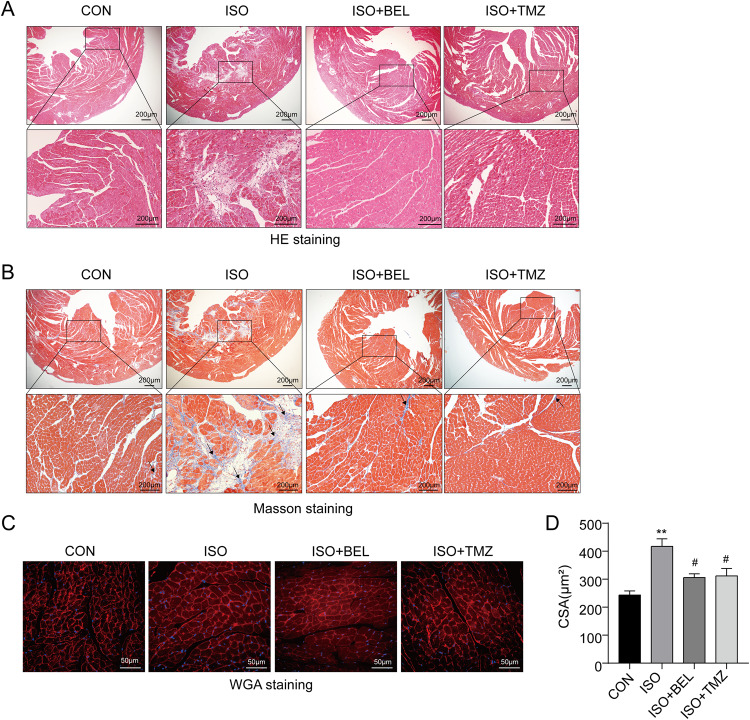
BEL alleviated cardiac histopathological change and cardiomyocyte hypertrophy in ISO-treated mice. **A** Representative images of HE staining of left ventricular tissue in different groups. **B** Representative images of Masson trichrome staining of left ventricular tissue in different groups. **C** Representative images of WGA staining of left ventricular tissue in different groups. **D** Bar graph showing the changes in cardiomyocyte cross-sectional area. Data are shown as the mean ± SEM. Significance: *p < 0.05 vs CON group; **p < 0.01 vs CON group; #*p* < 0.05 vs ISO group; ##*p* < 0.01 vs ISO group.

To evaluate the role of BEL on ISO-induced cardiac hypertrophy and concomitant fibrosis, cardiac histopathological changes were analysed. HE staining showed obvious disorder and an increased surface area of cardiomyocytes in the ISO group compared with the CON group, which indicated that the cardiac disease model had been successfully induced by ISO (Fig. 2A). These abnormalities were markedly ameliorated by treatment with BEL or TMZ. Furthermore, BEL markedly attenuated the ISO-induced cardiac fibrosis, which was marked by excessive collagen deposition as the blue staining areas in the Masson analyses (Fig. 2B). WGA staining is a suitable method for detection and quantification of cardiac hypertrophy and fibrosis in cardiac tissue [26, 27]. Here, the cross-sectional areas of cardiomyocytes, as the important indicators of cardiac hypertrophy, were evaluated by WGA staining (red) with DAPI (blue) for nucleus. The results indicated that the cross-sectional areas of cardiomyocytes were increased in the ISO group compared with the CON group (Fig. 2C, D). BEL suppressed the increase in the cardiomyocyte cross-sectional areas induced by ISO. These data illustrated that BEL ameliorated cardiac pathological changes and cardiomyocyte hypertrophy induced by ISO in mice.

### BEL ameliorated cardiac hypertrophy induced by ISO both in vitro and in vivo

**Fig. 3:**
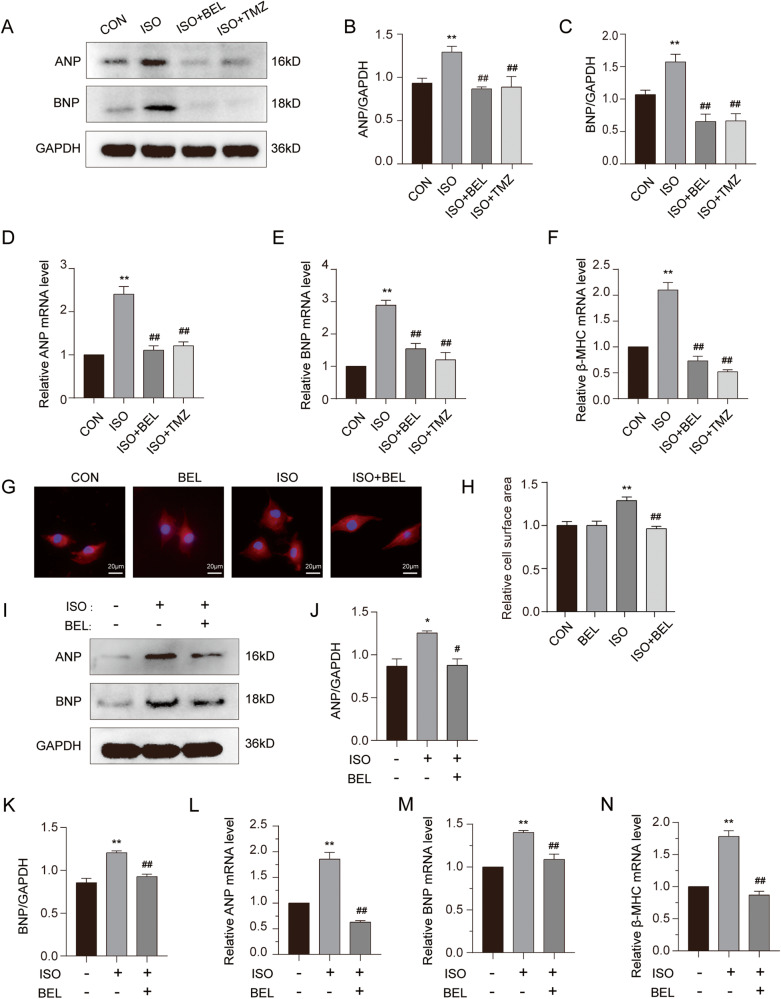
BEL reversed cardiomyocyte hypertrophy induced by ISO in vitro and in vivo. **A**–**C** Protein expression levels of ANP and BNP in cardiac tissues are detected via western blotting and quantitative analysis of the relative protein expression of ANP and BNP. GAPDH is used as a loading control. (*n* = 3). **D**–**F** The mRNA levels of ANP, BNP, and β-MHC in mice are measured by Quantified RT-PCR in each group, and relative mRNA levels normalise to GAPDH (*n* = 3). **G**–**H** The cell surface area was measured by rhodamine-phalloidin staining in H9C2 cells. **I**–**K** The protein level of ANP and BNP were determined in H9C2 cells by Western blot (*n* = 3). **L**–**N** The mRNA levels of ANP, BNP, and β-MHC are measured by Real-time PCR (*n* = 3). Data are shown as the mean ± SEM. Significance: *p < 0.05 vs CON group; **p < 0.01 vs CON group; #*p* < 0.05 vs ISO group; ##*p* < 0.01 vs ISO group.

Cardiac hypertrophy marker genes, including ANP and BNP were first measured by western blotting. The results showed that the levels of ANP and BNP proteins were significantly increased in the cardiac tissue of the mice treated with ISO compared to those in the CON group (Fig. 3A–C). These proteins were significantly decreased after BEL or TMZ treatment in the mice. Subsequently, we analysed the mRNA levels of these genes in the cardiac tissue of mice with or without ISO and BEL treatment via qRT-PCR. We found that both BEL and TMZ reduced the mRNA levels of ANP, BNP, and β-MHC, which had been increased by ISO treatment in mice (Fig. 3D–F). In summary, these results showed that BEL reduced both the mRNA and protein levels of cardiac hypertrophy marker genes that had been increased by ISO in mice.

To further explore the effect of BEL on ISO-induced cardiomyocyte hypertrophy in vitro, H9C2 cells were stained with phalloidin. As shown in Fig. 3G, H, the H9C2 cardiomyocyte surface area was markedly reduced with BEL after it had been increased by ISO treatment. Consistent with those of in vivo experiments, the protein levels of ANP and BNP were elevated after ISO incubation, and BEL inhibited the increase in these proteins in H9C2 cells (Fig. 3I–K). In addition, the qRT-PCR results showed that BEL downregulated the mRNA levels of ANP, BNP, and β-MHC, which had been increased by ISO treatment (Fig. 3L–N). These results illustrated that BEL inhibited the cardiomyocyte hypertrophy induced by ISO in both mice and H9C2 cells.

### BEL suppressed BRD4 during ISO-induced cardiac hypertrophy

**Fig. 4:**
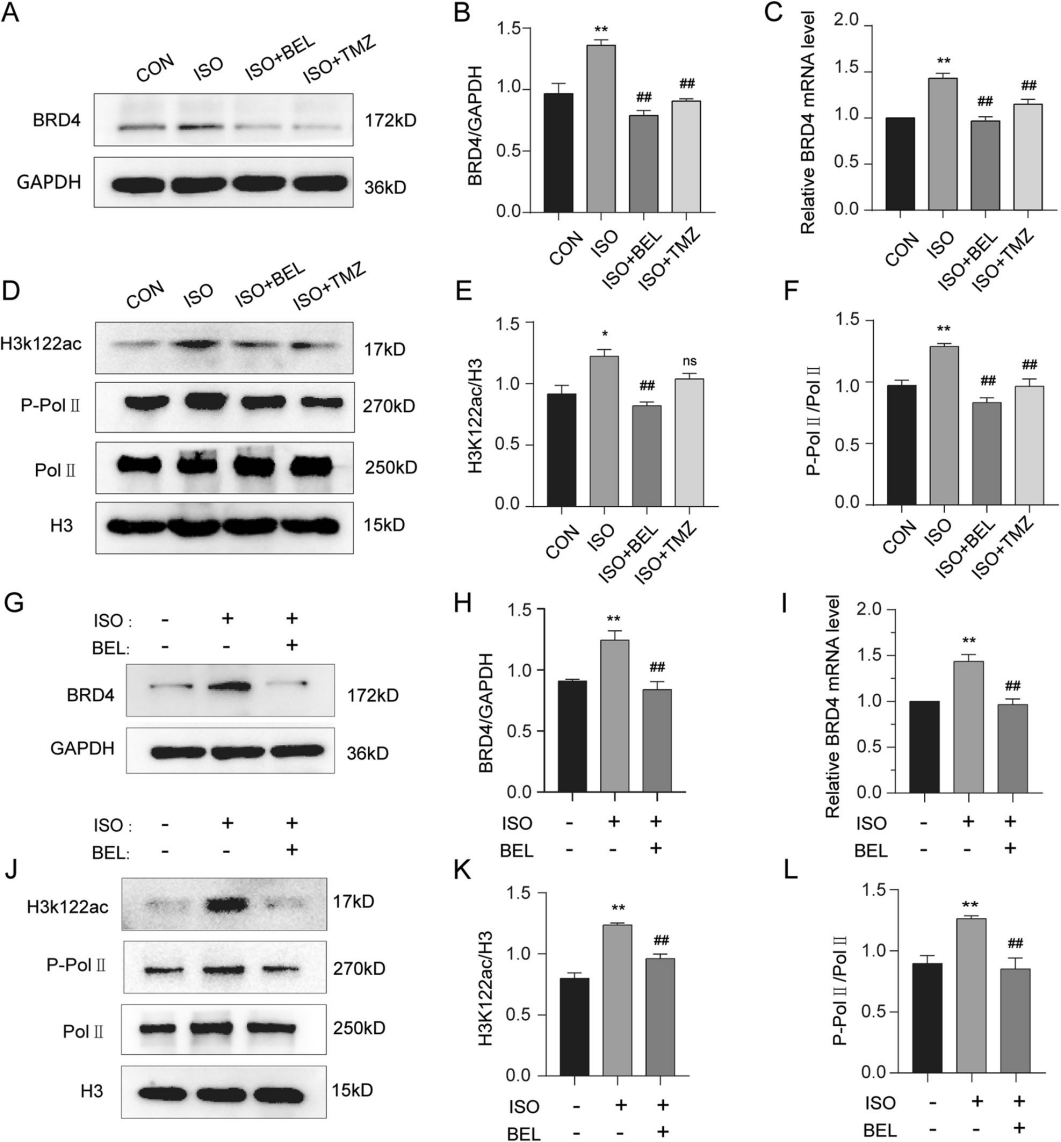
BEL decreased BRD4 protein and its functions induced by ISO. **A**, **B** The protein levels of BRD4 were determined by Western blot in cardiac tissues. GAPDH was used as a loading control (*n* = 3). **C** The mRNA levels of BRD4 were measured by qRT-PCR in cardiac tissues, and relative mRNA levels normalise to GAPDH (*n* = 3). **D**–**F** Western blot analysis was conducted to determine the phosphorylation of RNA Pol II, total expression and acetylation of K122 in cardiac tissues. H3 was used as a loading control (*n* = 3). **G**–**H** The protein levels of BRD4 were determined by Western blot in H9C2 cells. GAPDH was used as a loading control (*n* = 3). **I** The mRNA levels of BRD4 were measured by qRT-PCR, and relative mRNA levels normalise to GAPDH (*n* = 3). **J**–**L** Western blot was conducted to determine the phosphorylation of RNA Pol II, total H3 expression and acetylation of K122 in H9C2 cells (*n* = 3). Data are shown as the mean ± SEM. Significance: *p < 0.05 vs CON group; **p < 0.01 vs CON group; #*p* < 0.05 vs ISO group; ##*p* < 0.01 vs ISO group; ns no significant difference.

BRD4 is being increasingly acknowledged as a key epigenetic regulator during cardiac hypertrophy and fibrosis processes [28, 29]. First, western blot experiments were performed to analyse the regulatory function of BEL on BRD4 in cardiac tissues. BEL effectively decreased the BRD4 protein level induced by ISO (Fig. 4A, B). The mRNA level of BRD4 was also reduced by BEL in cardiac tissues treated with ISO (Fig. 4C). BRD4 acetylates H3K122, resulting in the nucleosome depolymerisation and transcription elongation, which depend on phosphorylation of RNA Pol II. In our experiments, ISO highly increased the acetylation of K122, which was substantially decreased by BEL but not by TMZ (Fig. 4D, E). In addition, BEL repressed the RNA Pol II phosphorylation induced by ISO, but the total protein level was not obviously changed (Fig. 4D, F).

Consistent with the results of in vivo experiments, ISO treatment upregulated both the protein and mRNA levels of BRD4, which was restored via BEL incubation in H9C2 cells (Fig. 4G–I). Furthermore, BEL inhibited the acetylation of H3K122 and RNA Pol II phosphorylation that had been induced by ISO treatment, with no evident change in H3 and RNA Pol II total protein levels (Fig. 4J–L). These data indicated that BEL inhibited BRD4 via H3K122ac and RNA Pol II activation during ISO-induced cardiac hypertrophy in vivo and in vitro.

### BEL ameliorated cardiac hypertrophy by suppressing BRD4

**Fig. 5:**
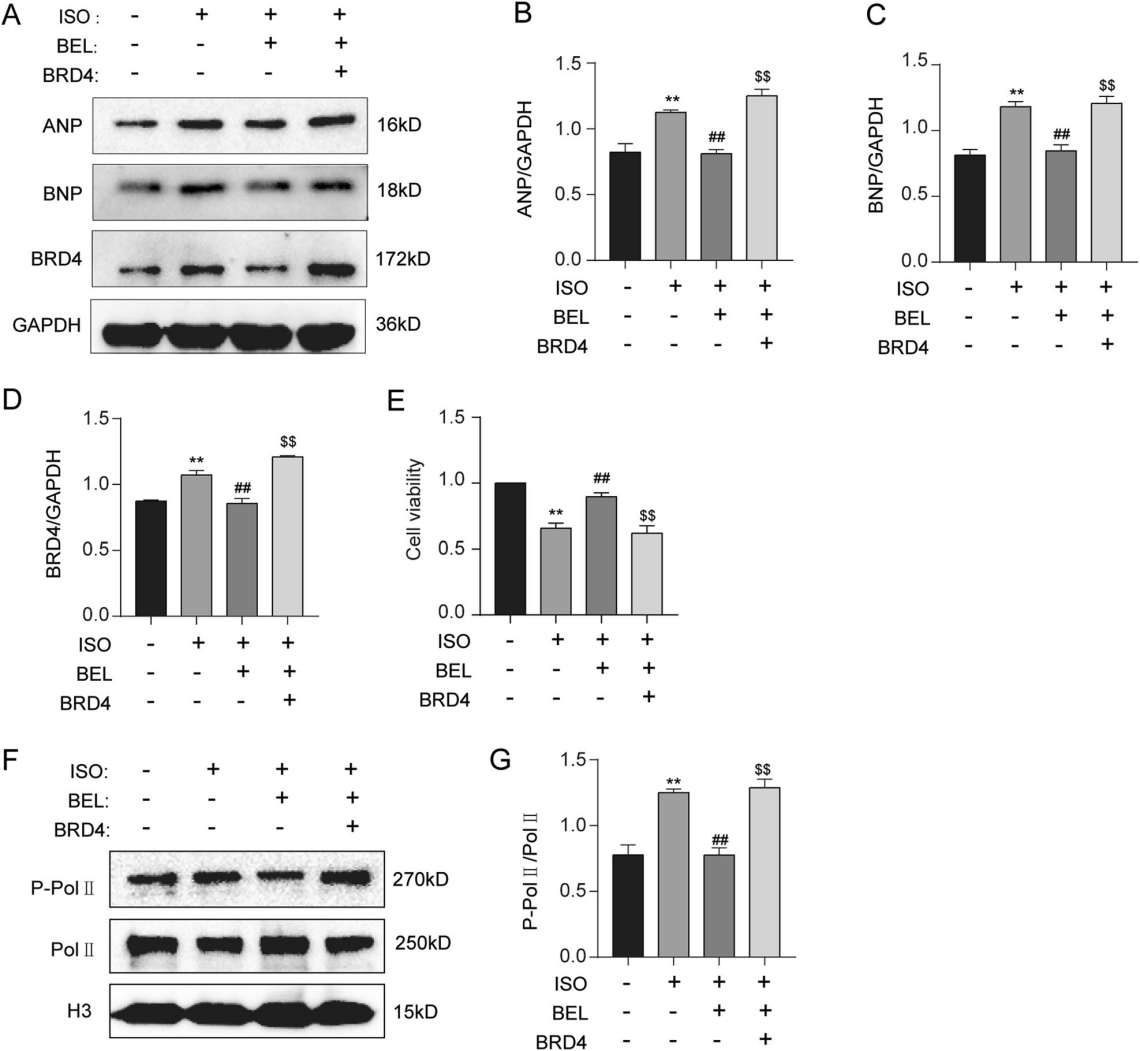
BEL inhibited ISO-induced elevation of BRD4 and its function. **A**–**D** Western blot analysis was performed to measure ANP, BNP and BRD4 expression levels in cells (*n* = 3). **E** CCK-8 assay was applied to investigate the cell viability in H9C2 cells. **F**, **G** Western blot analysis was performed to measure Pol II phosphorylation levels in H9C2 cells (*n* = 3). Data are shown as the mean ± SEM. Significance: *p < 0.05 vs CON group; **p < 0.01 vs CON group; #*p* < 0.05 vs ISO group; ##*p* < 0.01 vs ISO group; ^$^*p* < 0.05 vs ISO + BEL group ; ^$$^*p* < 0.01 vs ISO + BEL group.

To further investigate whether BEL ameliorated cardiac hypertrophy through BRD4, the experiment to compensate for the BEL-mediated reduction in BRD4 was performed. The results showed that BEL was not able to reduce ISO-induced ANP and BNP protein levels after the reversal of BRD4 protein, which illustrated that BEL ameliorated cardiac hypertrophy through its inhibitory function on BRD4 (Fig. 5A–D). In addition, BEL treatment restored the cell viability decreased by ISO, but this function of BEL was abrogated after recovery of the BRD4 protein level (Fig. 5E). Furthermore, the compensatory action for BRD4 blunted the BEL-induced reduction in Pol II phosphorylation levels in H9C2 cells pretreated with ISO (Fig. 5F, G). These results suggested that BEL exerted an anti- hypertrophic effect on cardiomyocytes in a BRD4-suppression-dependent manner.

### BEL inhibited Nox4 by suppressing BRD4 activity during ISO-induced cardiac hypertrophy

**Fig. 6:**
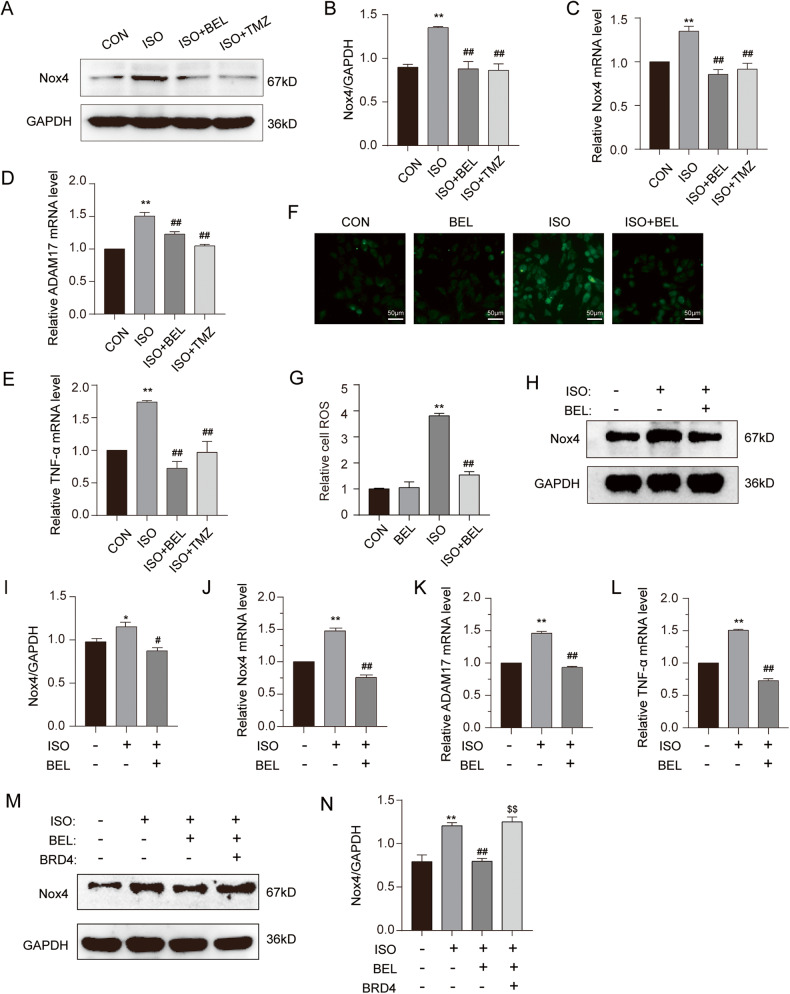
BEL inhibited the Nox4/ROS/ADAM17 pathway in ISO-induced cardiac hypertrophy. **A**, **B** The protein levels of Nox4 were determined by Western blot in cardiac tissues. GAPDH was used as a loading control (*n* = 3). **C** The mRNA levels of Nox4 were measured by qRT-PCR in cardiac tissues, and relative mRNA levels normalise to GAPDH (*n* = 3). **D**, **E** The mRNA levels of ADAM17 and TNF-a were measured by qRT-PCR in cardiac tissues, and relative mRNA levels normalise to GAPDH (*n* = 3). **F**, **G** DCFH-DA was used to measure the intracellular ROS. Confocal images showing ROS levels in H9C2 cells. **H**, **I** The protein levels of Nox4 were determined by Western blot in H9C2 cells. GAPDH was used as a loading control (*n* = 3). **J** The mRNA levels of Nox4 were measured by qRT-PCR in H9C2 cells, and relative mRNA levels normalise to GAPDH (*n* = 3). **K**, **L** The mRNA levels of ADAM17 and TNF-a were measured by qRT-PCR in H9C2 cells, and relative mRNA levels normalise to GAPDH (*n* = 3). **M**, **N** H9C2 cells were infected with adenovirus overexpressing BRD4 with or without BEL and ISO treatment. The protein levels of Nox4 were determined by Western blot (*n* = 3). Data are shown as the mean ± SEM. Significance: *p < 0.05 vs CON group; **p < 0.01 vs CON group; #*p* < 0.05 vs ISO group; ##*p* < 0.01 vs ISO group; ^$^*p* < 0.05 vs ISO + BEL group ; ^$$^*p* < 0.01 vs ISO + BEL group.

Considering that Nox4, a gene downstream of BRD4, is a major resource for ROS in the heart and can promote cardiac hypertrophy mediated through the Nox4/ROS/ADAM17 pathway [15, 16], the change in Nox4 protein and mRNA was analysed first. Both the protein and mRNA levels of Nox4 were decreased by BEL treatment of ISO-induced hypertrophy in cardiac tissues (Fig. 6A–C). We also measured the mRNA levels of ADAM17 and TNF-α, which were two marker genes of the Nox4/ROS/ADAM17 pathway. The results showed that BEL inhibited the upregulation of the mRNA levels induced by ISO (Fig. 6D, E). In addition, as shown in Fig. 6F, G, the results of the DCFH-DA assay showed that the ROS levels were significantly increased after ISO treatment, while BEL significantly decreased ROS fluorescence levels compared with those in the ISO group.

Furthermore, BEL inhibited the Nox4 protein and mRNA levels induced by ISO in H9C2 cells (Fig. 6H–J). The mRNA levels of ADAM17 and TNF-α were restored by BEL treatment after ISO induction (Fig. 6K, L). To further explore whether BEL represses Nox4 mediated through BRD4 during ISO-induced cardiac hypertrophy, BRD4 protein levels were restored. The results indicated that overexpression of BRD4 reversed the inhibition of Nox4 induced by BEL (Fig. 6M, N). These results suggested that BEL suppressed Nox4/ROS/ADAM17 pathway by inhibiting BRD4 in ISO-induced cardiac hypertrophy.

### BEL ameliorated ISO-induced cardiac hypertrophy in a BRD4/NOX4-signalling-dependent manner

**Fig. 7:**
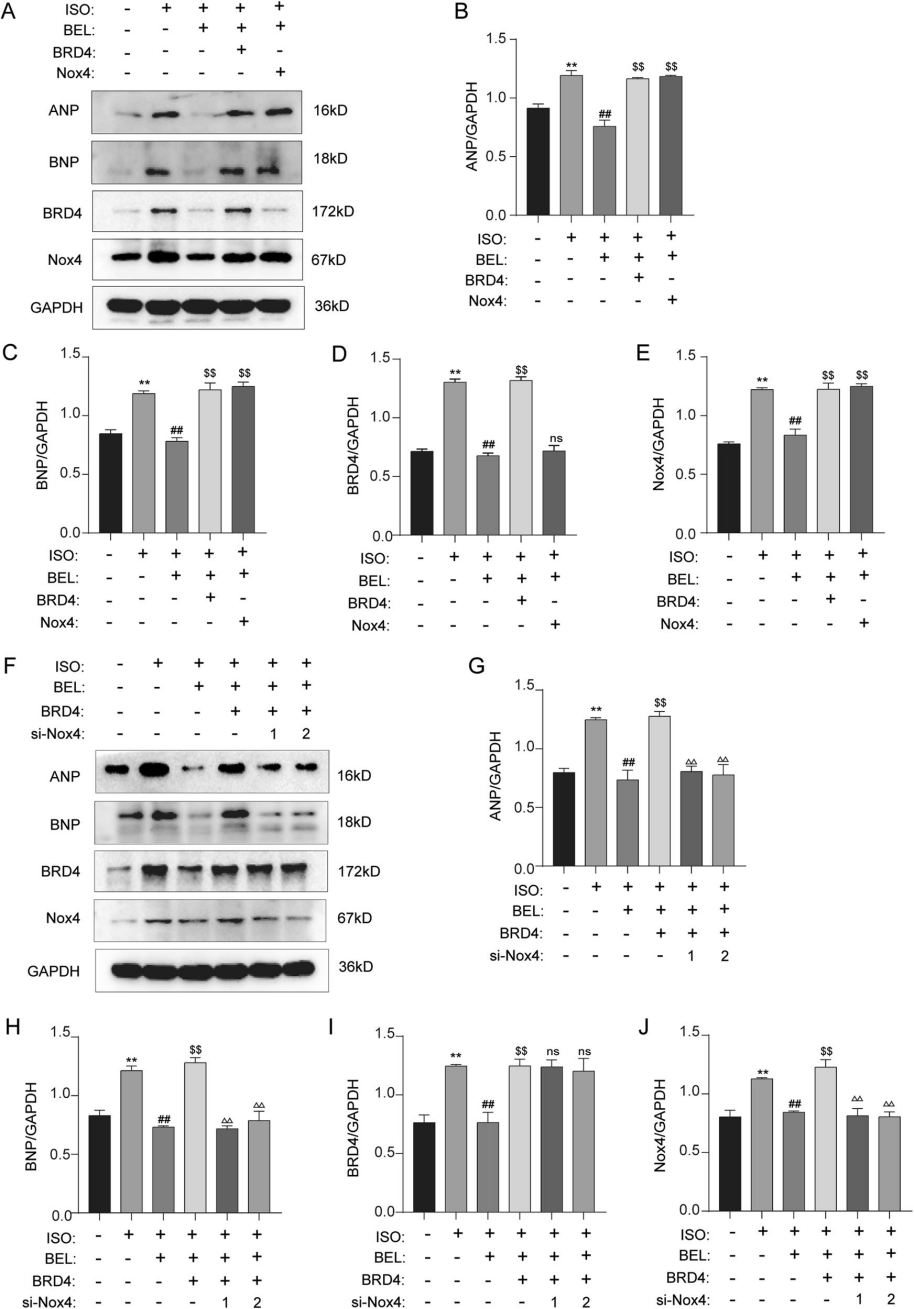
BEL inhibited ISO-induced cardiac hypertrophy via BRD4/NOX4. **A**–**E** H9C2 cells were transfected with Nox4 lentiviral and infected with adenovirus overexpressing BRD4 with or without BEL and ISO treatment. The protein levels of ANP, BNP, BRD4, and Nox4 were determined by Western blot. GAPDH was used as a loading control (*n* = 3). **F**–**J** H9C2 cells were transfected with si-Nox4 and infected with adenovirus overexpressing BRD4. Finally, cells were treated either with or without BEL and ISO. The protein levels of ANP, BNP, BRD4 and Nox4 were determined by Western blot. GAPDH was used as a loading control (*n* = 3). Data are shown as the mean ± SEM. Significance: *p < 0.05 vs CON group; **p < 0.01 vs CON group; #*p* < 0.05 vs ISO group; ##*p* < 0.01 vs ISO group; ^$^*p* < 0.05 vs ISO + BEL group ; ^$$^*p* < 0.01 vs ISO + BEL group; ^Δ^*p* < 0.05 vs ISO + BEL + BRD4 group; ^ΔΔ^*p* < 0.01 vs ISO + BEL + BRD4 group ; ns no significant difference.

We first investigated whether BRD4-mediated Nox4 inhibition contributes to BEL efficacy in ISO-induced cardiac hypertrophy. Thus, Nox4 expression recovery experiments were performed with H9C2 cells. As shown in Fig. 7A–E, compensation for both Nox4 and BRD4 blunted the antihypertrophic effects of BEL, as indicated by the increased protein levels of ANP and BNP in the cardiomyocytes. Moreover, although the upregulation of Nox4 was induced by BRD4 overexpression, increased Nox4 expression did not influence BRD4 protein levels. Therefore, Nox4 was unidirectionally downstream of BRD4 and mediated the antihypertrophic effects of BEL stimulated with ISO in H9C2 cells.

To further explore whether BEL/BRD4 ameliorated ISO-induced cardiac hypertrophy in a Nox4-dependent manner, Nox4 knockdown experiments were performed. As shown in Fig. 7F–J, BRD4 compensation blunted the antihypertrophic effects of BEL. However, the function of BRD4 compensation was not evident after Nox4 knockdown, proving that BEL/BRD4 mediated antihypertrophic effects through Nox4, which is downstream of BRD4. These results demonstrated that BRD4/NOX4 signalling mediated the antihypertrophic effects of BEL in the cardiomyocytes after ISO stimulation.

## Discussion

*G. acuta* plays a protective role in heart disease [22, 30]. Studies have focused on its functions in myocardial infarction through its inhibitory effect on classical inflammatory pathways [24]. In addition, our previous studies showed that *G. acuta* regulated cardiac remodelling mediated via the Notch and PI3K/Akt/FOXO1/3 pathways in whole tissue [22]. The heart comprises many cell types, such as fibroblasts and myocardial cells. However, the cell type (s) affected by *G. acuta* and those susceptible to drug-induced molecular changes have not been explained to date. In addition, the active compound in *G. acuta* that plays a role in these diseases has not been explicitly studied thus far. Therefore, studies into *G. acuta* to identify the effective components and its effect on various cell types in the heart are of great significance.

BEL is a natural xanthone that is abundant in *G. acuta* [31]. Studying the role of BEL in cardiovascular disease, previous studies reported that BEL attenuated cardiac fibrosis by inhibiting TGF-β1/Smad signalling in both heart tissue and cardiac fibroblasts [25]. Myocardial fibrosis usually occurs due to abnormalities in myocardial cells, such as cardiac hypertrophy [32]. However, the regulatory function of BEL on cardiomyocytes and cardiac hypertrophy remains largely unclear. Thus, our study focused on the function and regulatory mechanism of BEL in myocardial cells during cardiac hypertrophy. This study clarified that BEL inhibited pathological changes in ISO-induced myocardial hypertrophy and thus maintained cardiac function. Genes related to cardiac hypertrophy were notably regulated by BEL. Hence, as a potential therapeutic drug for cardiac hypertrophy, BEL may effectively alleviate the development of cardiac hypertrophy.

As a key potential therapeutic target, BRD4 has been implicated in many heart diseases, including pathological cardiac hypertrophy [28, 33, 34]. In our study, BEL reduced both the mRNA and protein levels of BRD4 during ISO-induced cardiac hypertrophy. The possible regulatory mechanism of BEL on BRD4 may be initiated before transcription. Presumably, BEL indirectly regulates BRD4 through intermediate molecules, thereby inhibiting BRD4 transcription and subsequent protein expression. Of course, it is also possible that BEL regulates BRD4 at the protein level, including it stability and activity. This outcome may be realised by either indirect regulation through other genes or direct interaction between BEL and the BRD4 protein. The regulatory mechanism of BRD4 by BEL may be intensively studied in the future.

Studies have shown that BRD4 is directly associated with the chromatin regulatory region of the NADPH oxidase subunit [35]. Furthermore, BET inhibition blocks the association of BRD4 with the Nox4 promoter [17, 36, 37]. In addition, BET inhibition significantly reduces oxidative stress [35]. BRD4 regulates downstream target gene transcription mainly through nucleosome depolymerisation, RNA Pol II phosphorylation, and transcription factor regulation, which depend on BRD4 acetylase and “acetylation reader” function [9, 10]. As the downstream target gene of BRD4, Nox4 was transcriptionally regulated by BEL through BRD4. BEL may have mediated this process through the three abovementioned regulatory mechanisms because H3K122ac abundance and the RNA Pol II phosphorylation level were changed during ISO and BEL treatment.

**Fig. 8:**
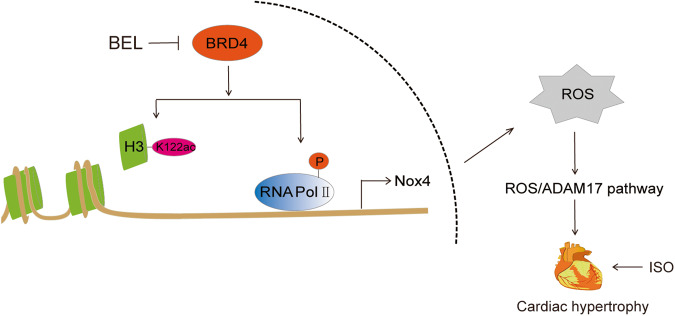
Schematic representation for BEL-mediated BRD4/Nox4/ROS signalling inhibiting during ISO-induced cardiac hypertrophy. BEL repressed BRD4 to reduce the ISO-induced acetylation of H3K122 and phosphorylation of RNA Pol II. Thus, the Nox4/ROS/ADAM17 signalling pathway was inhibited by BEL in a BRD4 dependent manner. Cardiac hypertrophy marker genes, including ANP, BNP, and β-MHC, were inhibited by BEL both in mice and in H9C2 cells. BEL alleviated cardiac hypertrophy and cardiac dysfunction via the BRD4/Nox4/ROS axes during ISO-induced cardiac hypertrophy.

Whether BRD4 is regulated by Nox4 via a positive or negative feedback loop remains unclear. In our experiments, overexpression and knockdown of Nox4 did not influence BRD4 protein levels after ISO-induced hypertrophy of H9C2 cells. In addition, when Nox4 was knocked down during ISO-induced hypertrophy, BEL did not reduce ANP and BNP protein levels after BRD4 expression recovery. These experiments illustrated that BRD4 acted upstream of Nox4 and that Nox4 did not affect BRD4 protein levels, although both molecules were regulated by BEL, inhibiting genes related to cardiac hypertrophy individually. Nox4 is a major enzyme that produces ROS, and oxidative stress involving the Nox4/ROS/ADAM17 signalling pathway was inseparable from the pathogenesis of cardiac hypertrophy [15, 16]. We found that BEL inhibited ISO-induced Nox4/ROS/ADAM17 pathway signalling by suppressing BRD4 and its transcriptional regulatory function. In summary, as shown in Fig. 8, BEL ameliorated cardiac hypertrophy and promoted cardiac function, making it a potential drug to target the BRD4/Nox4/ROS signalling pathway.

## Materials and methods

### Animals

Kunming male mice (18–22 g, 8–10 weeks old) were purchased from Liaoning Changsheng Biotechnology Co., Ltd. (China). Before the experiment, the mice were housed with controlled room temperature (21 ± 1 °C), humidity (55 ± 5%), and a 12/12 h light−dark cycle with free access to food and water. All experiments were performed in compliance with P.R. China legislation for the use and care of laboratory animals. All protocols for animal care were approved by the Institutional Animal Care and Use Committee of Hebei University of Chinese Medicine (no. DWLL2020018).

The mouse model of cardiac hypertrophy was established as previously described [38]. Briefly, ISO (5 mg/kg/day) was subcutaneously injected for seven consecutive days. The mice were randomly assigned to four groups (*n* = 10 per group): a control group (CON), model group (ISO), BEL intervention group (ISO + BEL, 30 mg/kg/day), and Trimetazidine (TMZ) intervention group (ISO + TMZ, 20 mg/kg/day). The drugs were purchased as follows: BEL from Chengdu Alfa Biotechnology, China; ISO from Tokyo Chemical Industry, Japan; and TMZ from Nanjing Zenkom Pharmaceutical Co., Ltd., China. The day after ISO injection, the mice were given the drug for 21 consecutive days. The mice in the control and model groups were given the same volume of 0.05% sodium cellulose carboxylate solvent by gavage. On Day 22, the mice were anaesthetised with 0.4% sodium phenobarbital and then sacrificed. The heart was fixed with 4% paraformaldehyde for histopathological analysis, and the remaining tissue was frozen and stored at −80 °C for later analyses. Animals and tissues were selected randomly via random number table for the experiments.

### Cell culture

H9C2 cell (rat cardiomyocyte cell line) was purchased from Procell (Wuhan, China). The cells after identifying were cultured in DMEM (Gibco, United States) supplemented with 10% foetal bovine serum (BI, Israel), penicillin (100 units/mL) and streptomycin (100 μg/mL) with 5% CO_2_ at 37 °C. BEL was dissolved in DMSO and diluted in buffer (the final DMSO concentration was 0.1%). For use in experiments, the cells were pretreated with DMSO or BEL (30 µM) for 0.5 h and stimulated with ISO (10 µM) for 12 h. BRD4 and Nox4 overexpression were induced via lentiviral and plasmid transduction. Nox4 was knocked down with siRNA. The siRNAs were designed and synthesised by GenePharma (Shanghai, China). A Lipofectamine 3000 transfection kit (Invitrogen) was used for siRNA transfection. After 24 h of gene overexpression, cells were pretreated either with or without 30 µM BEL for 0.5 h and ISO (10 µM) for 12 h. For the Nox4-knockdown experiment, cells were treated with BEL and ISO 48 h after siRNA transfection. Finally, the cells were collected for subsequent experiments. The siRNA sequences are listed in Supporting Information Table 1.

### Echocardiography and electrocardiograph measurements

Echocardiography and electrocardiograph measurements were performed on Day 21 of the experiment. Echocardiography was performed using the VEVO 2100 system (VisualSonics, Toronto, Ontario, Canada) to assess cardiac function. Mice were anaesthetised by inhalation of isoflurane (1.5%) and fixed in a supine position on a temperature-controlled operating table to keep the heart rate stable. The parasternal long-axis left ventricular function was evaluated by M-mode and B-mode imaging after hair removal with depilatory cream and application of ultrasound gel to the thoracic region. A standard lead II ECG was performed with a BL-420S system to evaluate the biological functions of mice in the experiment (Chengdu Taiman Software Co., Ltd.). Electrodes with needles were inserted into the subcutaneous tissue of the foot of an anaesthetised mouse, and the cardiac signals were continuously recorded for 60–120 s to identify ST segment changes.

### Morphological and pathological analysis

The middle segment of the left ventricle (LV) was fixed with 4% paraformaldehyde for pathological analysis. Fixed tissues were dehydrated, embedded in paraffin, and cut into 5-μm-thick sections. HE staining (Servicebio, China) and Masson’s trichrome staining (Servicebio, China) were performed to evaluate histopathological changes and collagen deposition. Photographs were taken with a light microscope (Leica, Germany). Myocyte hypertrophy was evaluated with wheat germ agglutinin (WGA) staining (Carlsbad, USA). The slides were photographed with a fluorescence microscope (Leica, Germany). These experiments were performed according to the instructions of the reagent manufacturers.

### Western blotting

Cells and heart tissues were lysed in RIPA buffer that included a protease inhibitor cocktail, phenylmethylsulfonyl fluoride, and phosphatase inhibitors. Tissue lysates were clarified via centrifugation at 15,000 × *g* for 15 min at 4 °C. The protein concentration was measured with a bicinchoninic acid protein assay kit (Solarbio, Wuhan, China). The sample proteins were separated by 10% SDS‒PAGE and transferred to PVDF membranes (Millipore, USA), which was blocked with 5% nonfat milk for 90 min at room temperature. The membranes were individually incubated overnight with antibodies at 4 °C and then with the appropriate secondary antibodies for 60 min at room temperature.

Primary antibodies against BRD4 (Abcam; ab75898; 1:1000), ANP (Abcam; ab225844; 1:1000), BNP (Abcam; ab239510; 1:1000), histone H3 (Abcam; ab176842; 1:1000), acetyl histone H3K122 (Abcam; ab33309; 1:1000), RNA Pol II phosphorylation on serine 2 (Abcam; ab193468; 1:5000), RNA Pol II (CST; 2629; 1:1000), Nox4 (Proteintech, 14347-1-AP; 1:1000), and GAPDH (Proteintech, 60004-1-Ig; 1:10000) were used in this study. HRP-conjugated anti-rabbit IgG (cat. no. ZB-2301) and anti-mouse IgG (cat. ZB-2305) were purchased from the ZSGB Bioengineering Institute (Beijing, China).

Immunoreactive bands were detected using an ECL chemiluminescent substrate kit (NCM Biotech, P10300) and visualised using a chemiluminescence imager OmegaLum W (Minneapolis, MN, USA). Protein expression was quantified with ImageJ software (National Institutes of Health, Bethesda, MD) and normalised to that of GAPDH.

### Real-time fluorescent quantitative PCR (qRT‒PCR) analysis

Total RNA from mouse heart tissues and cells was extracted with an Eastep Super Total RNA Extraction Kit (Omega Bio-Tek), and cDNA was synthesised with a reverse transcription kit (Generay Biotech, Shanghai, China) according to the respective manufacturer’s instructions. Real-time PCR was performed using quantitative SYBR green PCR mix (Bio–Rad). The levels of target mRNAs were normalised to the level of GAPDH expression following the 2−ΔΔCt method. All primers used in this study are listed in Supporting Information Table 2.

### Cell viability

Cell viability was evaluated by Cell Counting Kit-8 (CCK-8) (MedChemExpress, United States) according to the manufacturer’s instructions. H9C2 cells were seeded in 96‐well plates and grown to 70–80% confluence. Then, 10 μL of CCK-8 solution was added to each well and incubated for 1 h at 37 °C. The OD values were measured at 450 nm with a microplate reader (Thermo Fisher Scientific, United States).

### Cell surface area analysis

H9C2 cells grown on coverslips were fixed with 4% paraformaldehyde for 10 min at room temperature and permeabilized with 0.1% Triton X-100 for 10 min. Then, the cells were incubated with 0.1% rhodamine-phalloidin (Invitrogen, Carlsbad, CA, USA) for 1 h. After washing with PBS, the cells were then incubated with DAPI (Servicebio, Wuhan, China). The slides were visualised with a fluorescence microscope (Leica, Germany).

### ROS level measurement

Intracellular ROS levels were determined with a ROS detection kit (Beyotime, Jiangsu, China). Briefly, cells were treated with 10 µM DCFH-DA dissolved in DMEM for 20 min at 37 °C. Then, they were washed three times with phosphate-buffered saline (PBS). Finally, the ROS levels were measured with a fluorescence microscope via green fluorescence (Leica, Germany).

### Statistical analysis

All data were analysed using SPSS 21.0 statistical software and expressed as the mean ± standard error of the mean. The significance of differences among multiple groups was tested with one-way ANOVA with Tukey’s post hoc test. In all cases, differences with a *P* value < 0.05 were considered to be statistically significant.

## Supplementary information

Supplementary Table 1: The siRNA sequences of Nox4 for the knockdown experiment in the manuscript.

Supplementary Table 2: The primer sequences used for the qRT-PCR experiments in the manuscript.

Original Data File

## Data Availability

The datasets used during the study are available from the corresponding author on request.
