# Supplementary material for: Bellidifolin ameliorates isoprenaline-induced cardiac hypertrophy by the Nox4/ROS signalling pathway through inhibiting BRD4
Source: Cell Death Discov. 2023 Aug 1;9:279. doi: 10.1038/s41420-023-01563-2 (PMC10394041; doi:10.1038/s41420-023-01563-2)
Supplement: Supplementary file 1 — Supplementary Table 1: The siRNA sequences of Nox4 for the knockdown experiment in the manuscript. [file 41420_2023_1563_MOESM1_ESM.docx]

Supplementary Table 1: The siRNA sequences of Nox4 for the knockdown experiment in the manuscript.

| siRNA sequences | Sequences 5–3 |
| --- | --- |
| siNox4-1-F | GGCUUGUUGAAGUAUCAAATT |
| siNox4-1-R | UUUGAUACUUCAACAAGCCTT |
| siNox4-2-F | GCCCUUCAUUCAAUCUAGATT |
| siNox4-2-R | UCUAGAUUGAAUGAAGGGCTT |

Table 1 siRNA sequences
