# Supplementary material for: Bellidifolin ameliorates isoprenaline-induced cardiac hypertrophy by the Nox4/ROS signalling pathway through inhibiting BRD4
Source: Cell Death Discov. 2023 Aug 1;9:279. doi: 10.1038/s41420-023-01563-2 (PMC10394041; doi:10.1038/s41420-023-01563-2)
Supplement: Supplementary file 2 — Supplementary Table 2: The primer sequences used for the qRT-PCR experiments in the manuscript. [file 41420_2023_1563_MOESM2_ESM.docx]

Supplementary Table 2: The primer sequences used for the qRT-PCR experiments in the manuscript.

Table 2 Primers used for the genes qRT-PCR design

| Primer (mice) | Sequences 5–3 |
| --- | --- |
| ANP (m) F | TGGGCTTCTTCCTCGTCTTG |
| ANP (m) R | TCTAGCAGGTTCTTGAAATCCATC |
| BNP (m) F | GCTGCTGGAGCTGATAAGAGAA |
| BNP (m) R | CGATCCGGTCTATCTTGTGCC |
| β-MHC (m) F | ACTGTCAACACTAAGAGGGTCA |
| β-MHC(m)R | TTGGATGATTTGATCTTCCAGGG |
| BRD4 (m) F | GTGAGAAGCTAGGCCGTGTAG |
| BRD4 (m) R | AGGCAGGACCTGTTTCAGAGT |
| NOX4 (m) F | CCGGACAGTCCTGGCTTATC |
| NOX4 (m) R | TGCTTTTATCCAACAATCTTCT |
| Adam17 (m) F | GTGCTGGGAAGATCACCTCC |
| Adam17 (m) R | CACCACCTCTCTGGGAAACC |
| TNF-α (m) F | ATGTCTCAGCCTCTTCTCATTC |
| TNF-α (m) R | GCTTGTCACTCGAATTTTGAGA |
| GAPDH (m) F | AGGTCGGTGTGAACGGATTTG |
| GAPDH (m) R | TGTAGACCATGTAGTTGAGGTCA |

| Primer (Rat) | Sequences 5–3 |
| --- | --- |
| ANP (Rat) F | AGCGAGCAGACCGATGAAG |
| ANP (Rat) R | AGCCCTCAGTTTGCTTTTCA |
| BNP (Rat) F | TTTGGGCAGAAGATAGACCG |
| BNP (Rat) R | AGAAGAGCCGCAGGCAGAG |
| β-MHC (Rat) F | TGGAGCTGATGCACCTGTAG |
| β-MHC (Rat) R | ACTTCGTCTCATTGGGGATG |
| BRD4 (Rat) F | ACAGCCCCAACAGAACAAAC |
| BRD4 (Rat) R | GCTGGTTCCTTCTTGCTCAC |
| NOX4 (Rat) F | TGCATGGTGGTGGTATTGTTCCTC |
| NOX4 (Rat) R | AGCAGCAGCAGCATGTAGAAGAC |
| Adam17 (Rat) F | GTGAGCAGTTTCTCGAACGC |
| Adam17 (Rat) R | AGCTTCTCAAGTCGCAGGTG |
| TNF-a (Rat) F | TGGCGTGTTCATCCGTTCTC |
| TNF-a (Rat) R | CCCAGAGCCACAATTCCCTT |
| GAPDH (Rat) F | GGTGGTCATATGACAAAACTTGAAGAG |
| GAPDH (Rat) R | CACCCTGTTGCTGTAGCCAAA |
