# Supplementary material for: Bellidifolin ameliorates isoprenaline-induced cardiac hypertrophy by the Nox4/ROS signalling pathway through inhibiting BRD4
Source: Cell Death Discov. 2023 Aug 1;9:279. doi: 10.1038/s41420-023-01563-2 (PMC10394041; doi:10.1038/s41420-023-01563-2)

Original data of western blotting:

Supplementary figures: Western blotting in the manuscript was performed for three times. The original data of western blotting with three times were shown for Fig. 3A, Fig. 3J, Fig. 4A, Fig. 4D, Fig. 5A, Fig. 5F, Fig. 6A, Fig. 6H, Fig. 6M, Fig. 7A and Fig. 7F of the manuscript.


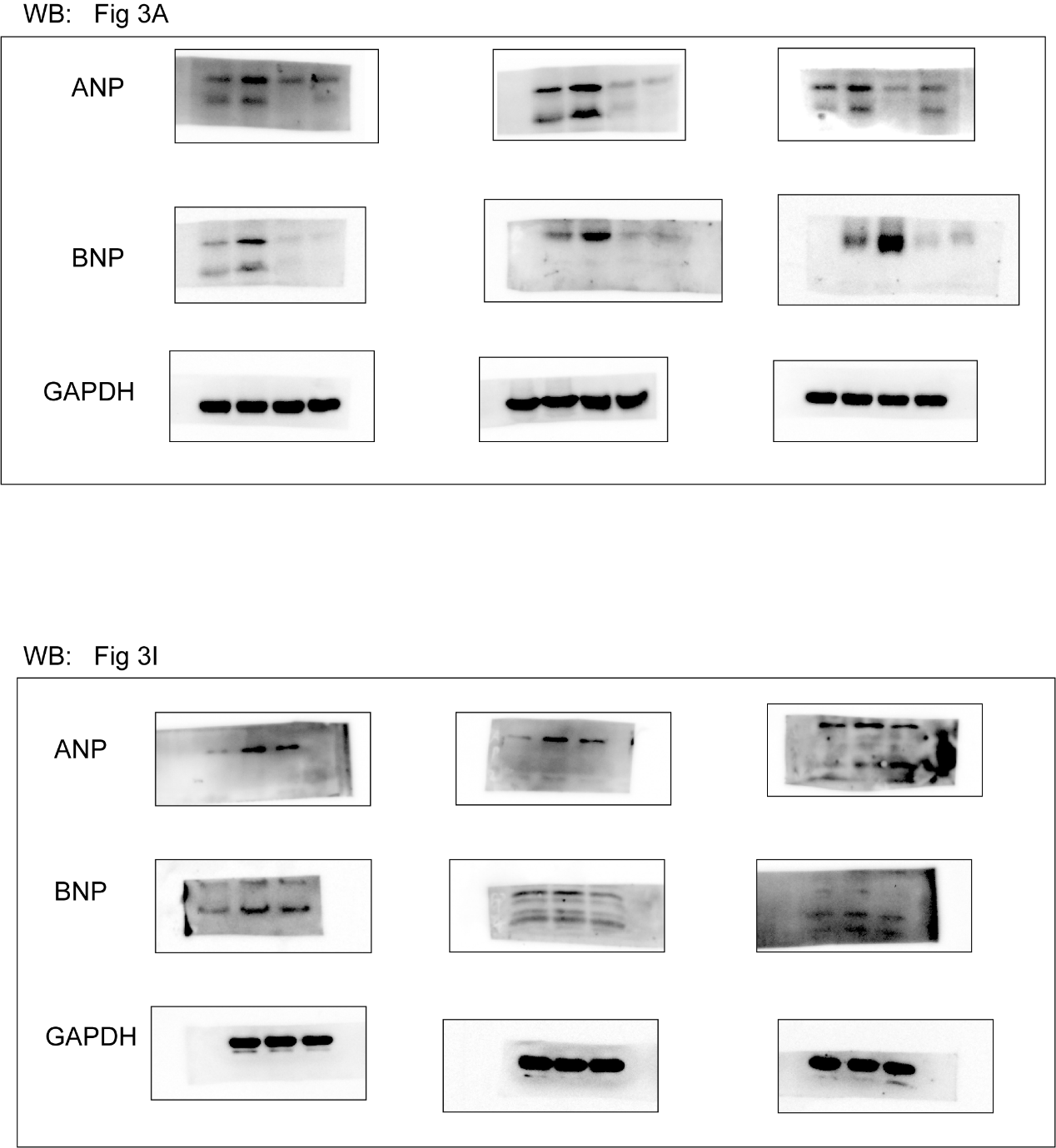


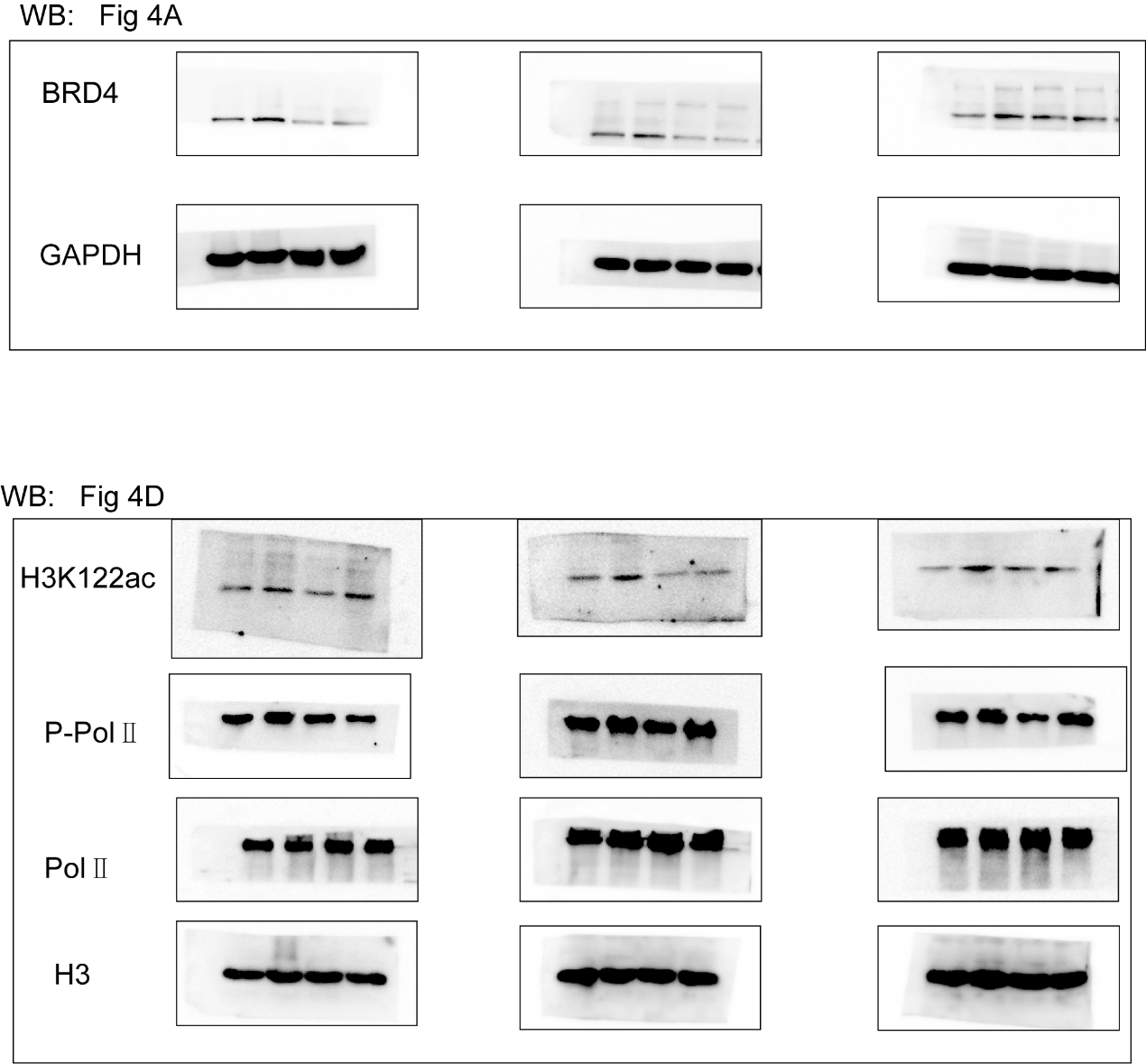


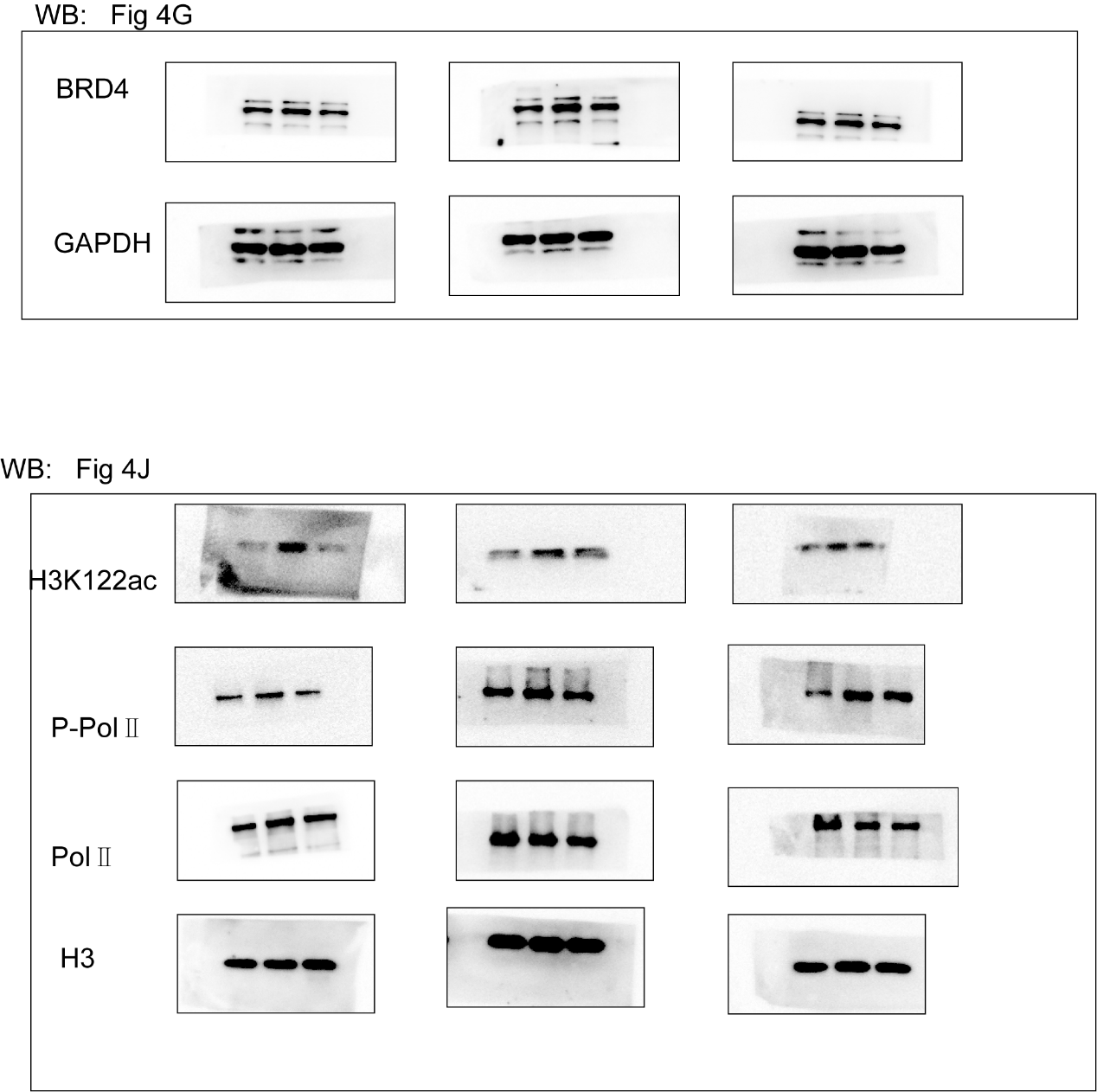


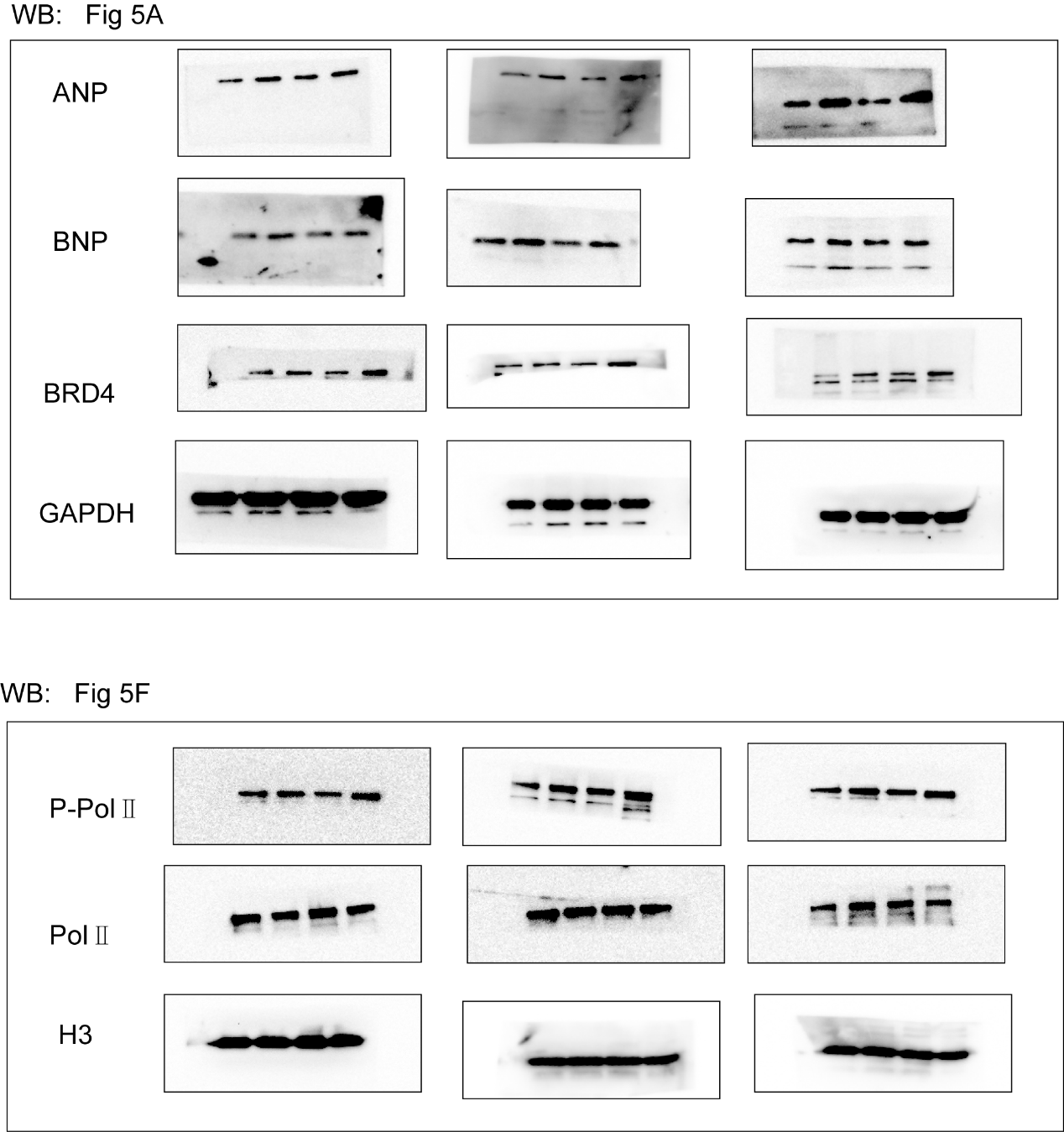

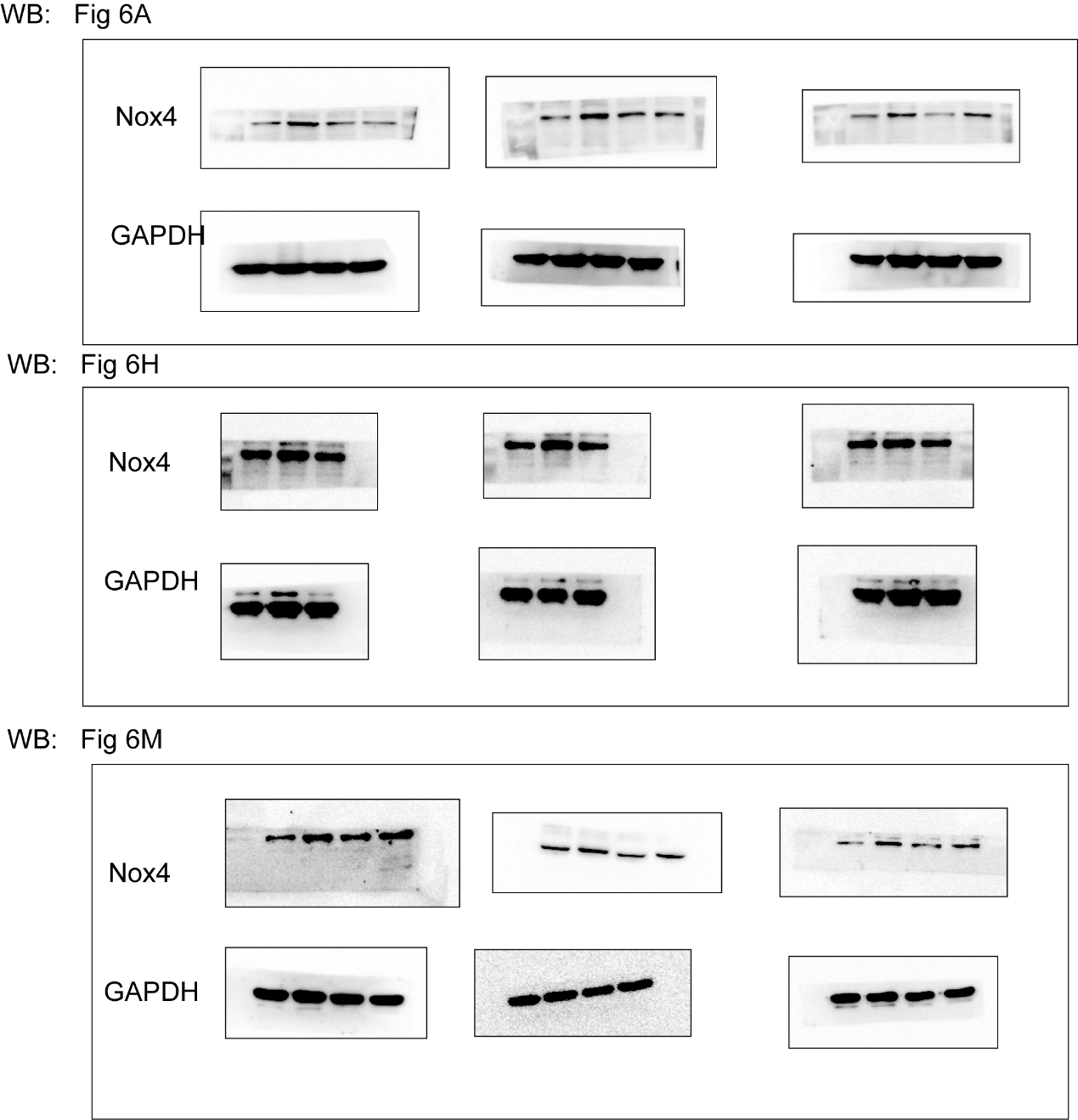

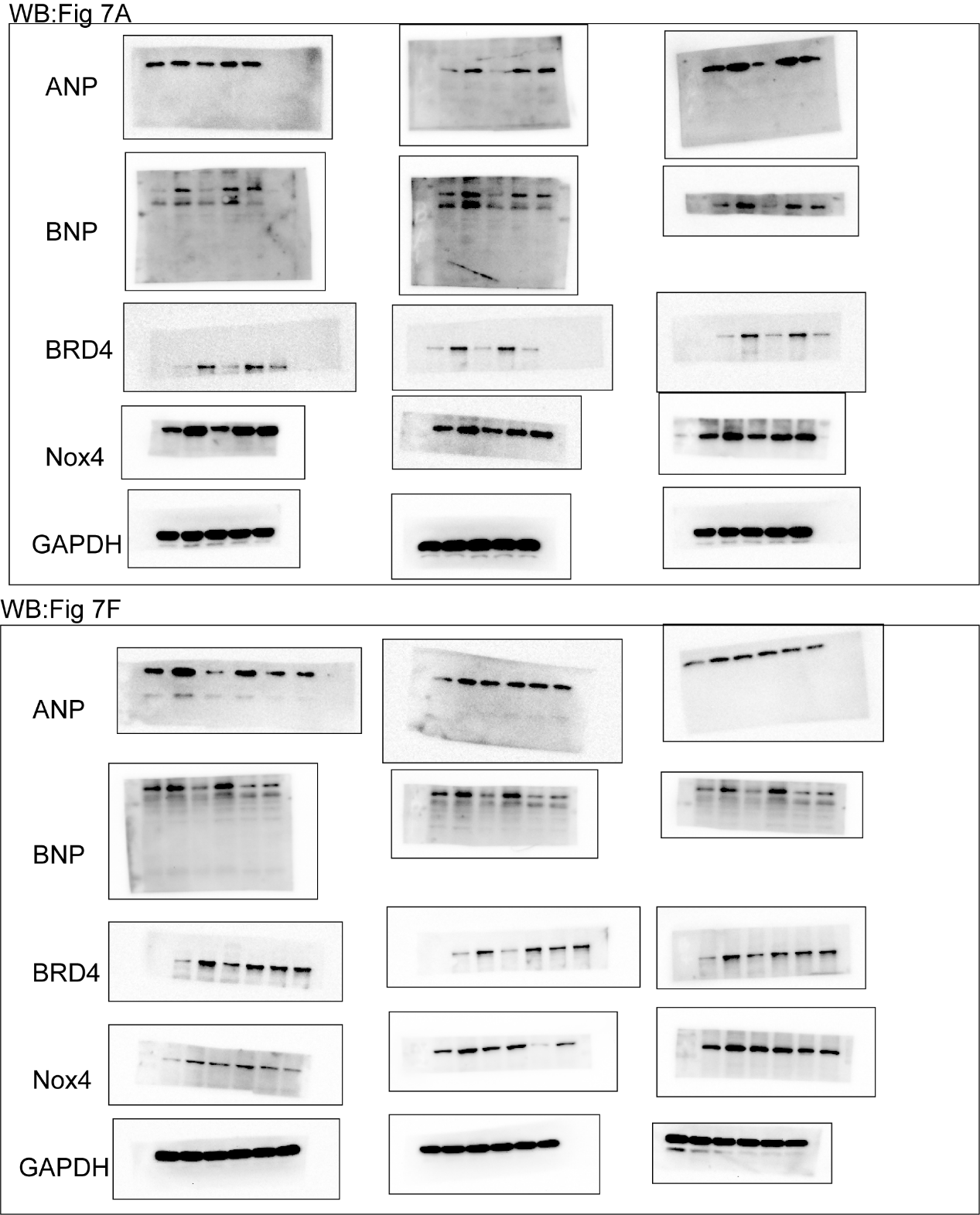

Supplement: Supplementary file 3 — Original Data File [file 41420_2023_1563_MOESM3_ESM.docx]
